# Association between ABO blood groups and postoperative pain in children after adenotonsillectomy: a prospective cohort study

**DOI:** 10.1186/s12871-022-01953-6

**Published:** 2022-12-28

**Authors:** Longyuan Shen, Jianwen Wu, Quansheng Xiao, Mingyan Hong, Shengzhao Wang, Jingti Wang, Qichen Luo, Xiongda Lin, Jianfeng Lian, Yibin Xu, Guoqing Guo, Junzheng Wu

**Affiliations:** 1 Department of Anesthesiology, Quanzhou Women’s and Children’s Hospital, Fujian Province, Quanzhou, China; 2 Department of Otolaryngology, Quanzhou Women’s and Children’s Hospital, Fujian Province, Quanzhou, China; 3 Department of Anesthesiology, Cincinnati Children’s Hospital Medical Center, Cincinnati, OH USA

**Keywords:** ABO blood groups, Postoperative pain, Paediatrics, Adenotonsillectomy

## Abstract

**Background:**

It has been known that ABO blood groups are linked to the phenotypes of certain diseases; however, and the relationship between ABO blood groups and postoperative pain have not been extensively studied, especially in children. This study was to investigate whether there would be an association between the four major ABO blood groups and postoperative pain, as indicated by the differences in pain scores and rescue fentanyl requirements among blood groups in children after adenotonsillectomy.

**Methods:**

A total of 124 children, aged 3–7 years, ASA I or II, and undergoing elective adenotonsillectomy were enrolled in the study. Postoperative pain was evaluated using the Children’s Hospital of Eastern Ontario Pain Scale (CHEOPS) and the rescue fentanyl requirement in post anesthesia care unit (PACU) was analyzed. Pediatric Anesthesia Emergence Delirium (PAED) score and the duration of PACU were recorded. The postoperative nausea and vomiting (PONV) within 24 h were documented.

**Results:**

Among four blood type groups, no significant differences were observed regarding surgery time, and the gaps of fentanyl given at the anesthesia induction and the first rescue fentanyl injection in PACU. However, patients from AB and B blood groups had significantly higher pain score at initial CHEOPS assessment and consequently, higher consumption of rescue fentanyl during PACU stay. A significantly higher percentage of patients had received > 1 μg/kg rescue fentanyl. Higher PAED scores were also observed in AB and B blood groups.

**Conclusion:**

Paediatric patients with AB and B blood type had higher postoperative CHEOPS pain score and required significantly more fentanyl for pain control than those with A and O blood type after T&A. The initial scores of PAED in patients with AB and B blood type were also higher than that in patients with A and O blood type.

**Supplementary Information:**

The online version contains supplementary material available at 10.1186/s12871-022-01953-6.

## Background

Clinically, the pain intensity and the perception among the individuals in the same surgical setting vary dramatically [1]. The differences in expression and reaction to pain could be influenced by factors like genetic, developmental, familial, psychological, social and cultural variables [2, 3]. The growing evidences indicate that the heterozygous DNA elements have strongly linked to the substantial inter-individual variation in pain sensitivity and susceptibility and associated with the development of certain pain disorders, and the way of response to analgesics. However, the perception of physiological pain hasn’t been pin-pointed to any specific DNA variants yet. As we know, ABO blood type is one of the important genetic phenotypes among all DNA variants, and it might have an association with pain sensitivity and susceptibility. It has been known that ABO blood types are strongly correlated with certain medical conditions, such as cancer [4], cardiovascular disease [5], and diabetes [6]. Previous studies have found that ABO blood type has played some role in anesthesia and pain expression [1, 7]. Du et al. [7] revealed that under general anesthesia with propofol, patients with blood type B had higher mean arterial pressure (MAP) and heart rate (HR), while patients with blood type A had higher BIS readings. Another study found that participants with blood type B showed the highest pressure-induced pain threshold, while patients with AB blood type showed the lowest (most sensitive to pain) [1]. Mohammad et al. [8] found a possible association between opioid addiction and human blood type. However, studies regarding the association between ABO blood group and pain remain limited; particularly, no prospective study been designed to investigate the relationship between ABO blood type and perioperative pain, especially in paediatric patients.

Adenoid and tonsillar hypertrophy are common in children and are often as the cause of severe obstructive sleep apnea syndrome (OSAS) [9]. Adenotonsillectomy (T&A) is one of the most commonly performed operations in children [10]. Most children will develop moderate to severe postoperative pain, and the pharmacological approaches, primarily opioids, have been the mainstream pain management. Even with multiple choices of analgesics available, the unsatisfactory pain control of post-adenotonsillectomy is still a vexing issue for clinicians and patients. Therefore, more studies for better understanding the pain mechanisms and effective management of postoperative pain in children would have more clinical significance.

The aim of our study was to investigate whether there was an association between ABO blood types and postoperative pain, as indicated by pain scores and the fentanyl consumption in children underwent T&A.

## Methods

This study (registration #1,900,022,743 on chictr.org.cn) was conducted between May 2019 and November 2020 after approval by the Ethics Committee of QuanZhou Women's and Children's Hospital (the approval number of the ethics committee: 2019–02).The risk and benefits of the study were discussed with families and subsequently, written informed consent was obtained from the parents or legal guardians. All methods were performed in accordance with the relevant guidelines and regulations. All participants followed a standard perioperative care protocol.

Patients aged 3–7 years, ASA I or II, and scheduled for elective T&A were enrolled. All children presented a history of parent-reported snoring and restless sleep, and adenoid and tonsillar hypertrophy were diagnosed by an otolaryngologist upon physical examination at physician’s office (score ≥ 1 over a standardized scale of 0–4). The exclusion criteria were: ongoing adenoid and tonsil inflammation, history of previous surgery, craniofacial deformities, mental retardation, and body mass index of > 22 kg/m^2^, a history of bronchial asthma, and recent opioid use.

Patients were admitted to hospital on the day before surgery. In the surgical ward, intravenous (IV) access was established and blood was drawn for blood typing. On the day of surgery, the patients were transferred to the surgical waiting area. The maintenance rate of Lactated-Ringer solution was initiated. No pre-medications were administered by study protocol. Continuous pulse oximetry and HR monitoring was started in holding area, and patient was induced with propofol 2 mg/kg. Patients were moved to OR after falling into sleep, and the standard monitors were place immediately. The entire process of induction and transport to OR was completed in less than 2 min.Standard monitoring was applied (pulse oximetry, electrocardiography, noninvasive blood pressure) and 2 μg/kg fentanyl and 0.6 mg/kg rocuronium were administered to finish the anesthesia induction. The patient was manually ventilated for 2 min with oxygen and sevoflurane, and endotracheal intubation was performed. Anesthesia was maintained with 2%-3.5% sevoflurane and 60% nitrous oxide in oxygen, and ETCO_2_ concentrations were maintained at 35–45 mmHg during surgery with pressure-controlled ventilation mode. All children received IV dexamethasone 0.15 mg/kg (maximum 10 mg) and ondansetron 0.1 mg/kg at the end of anesthesia induction and just before starting surgery. No additional fentanyl or other pain medications were administered during the surgery. A radiofrequency ablation surgery were performed in all patients by attending otolaryngologist. HR was recorded continuously and noninvasive MAP was measured every 5 min. Data of vital signs were collected at following time points: baseline (T0), after anesthesia induction (T1), at the beginning of the operation (T2), 10 min (T3) and 20 min (T4) after the surgery started, at the end of the surgery (T5), and after tracheal tube removal (T6).

At the end of surgery, the tracheal tube was removed when the extubation criteria had been met (spontaneous and regular breathing, voluntary eye opening, purposeful body movement, and ETsevo concentration < 0.3%), and patients were transferred to the post anesthesia care unit (PACU). Once the patient had voluntary response (body movement, eye opening, vocal response), the postoperative Children’s Hospital of Eastern Ontario Pain Scale (CHEOPS) score was evaluated immediately by a designated anesthetist who was blinded to the patient’s grouping. The pain score was re-assessed every 5 min.

The CHEOPS is an observational tool for measuring postoperative pain in children aged 1–7 years and has six categories of pain behavior: cry, facial, verbal, torso, touch, and legs. The score ranges from 0 to 2 or 1 to 3 which are assigned to each activity, and the total score ranges between 4 and 13, with a score of 4 described as no pain and 13 described as the worst pain. Pain is rated as mild, moderate, or severe when the score falls in the range of 5–7, 8–10, and 11–13, respectively. In this study, a score of ≥ 6 was defined as the presence of uncomfortable pain, which necessitated administration of a bolus of IV fentanyl 0.5 μg/kg as rescue analgesic. If the CHEOPS score ≥ 6 persisted, fentanyl injection was repeated every 5 min for a maximum of four times (total 2 μg/kg). Beyond that, 50 μg/kg morphine was administered if the maximally allowed dosage of fentanyl was reached and the patient’s pain was remained at uncomfortable level. IV morphine injection was repeated once (maximal 100 μg/kg). Additional analgesic administration could be used at the anesthesiologist’s discretion. Patients’ vital signs were continuously monitored in the recovery room, and oxygen was supplied via a face mask if the child’s SpO_2_ was < 95%. The Pediatric Anesthesia Emergence Delirium (PAED) score was recorded at the same time points as the CHEOPS assessment. Postoperative nausea/vomiting (PONV) and other adverse events, such as airway obstruction or laryngospasm, were also recorded in the recovery room. Patients were transferred to the surgical ward once the discharge criteria had been met (Aldrete score ≥ 9, CHEOPS score < 6).

The primary outcome was postoperative pain indicated by the pain score and total fentanyl consumption among the four major ABO blood groups. The secondary outcomes included PAED scores, incidence of PONV, hypoxia, and other potential complications (laryngospasm) recorded in the recovery room.

### Statistical analysis

Statistical analysis was performed using the SPSS 24.0 Normally distributed data were expressed using mean ± standard deviation and were analyzed using variance analysis. On the other hand, non-normally distributed data were expressed as median with interquartile range (IQR), and analyzed using the Kruskal–Wallis test. Enumeration data were expressed as percentages and analyzed using the chi-square test. Statistical significance was set at *P* < 0.05.

## Results

A total of 124 children were enrolled and had complete data collected during the study. Table 1 shows the number of patients in each group and the other demographic data.

**Table 1:** Characteristics of participants

Blood type	A	B	O	AB	F/χ^2^	*p* value
Cases Age	33 4.88 ± 1.17	30 4.63 ± 1.10	35 4.94 ± 1.28	26 4.88 ± 1.14	0.420	0.739
M / F	18/15	18/12	22/13	16/10	0.547	0.908
Wt(kg)	19.45 ± 5.03	19.06 ± 4.02	19.12 ± 4..23	17.71 ± 2.34	0.967	0.411
Surgical time (min) Induction fentanyl to rescue fentanyl (min)	34.7 ± 6.1 39.3 ± 8.1	35.8 ± 7.1 42.1 ± 8.9	34.7 ± 5.3 40.4 ± 9.4	34.8 ± 6.0 39.8 ± 6.9	0.486 0.634	0.693 0.595

**Fig. 1:**
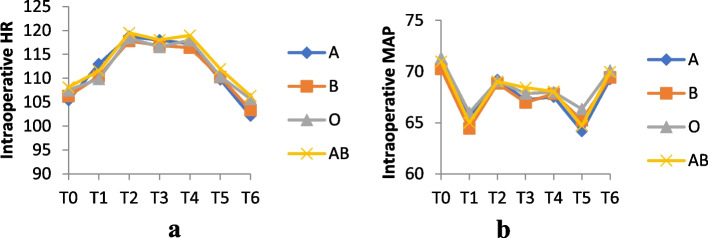
Intraoperative HR (**a**) and intraoperative MAP (**b**) at each time point. No significant differences of HR and MAP at each time point between groups were found (*P* > 0.05)

No significant differences were observed among groups regarding the age, sex ratio, weight, operation time, and the time span for fentanyl given at anesthesia induction up until the first rescue fentanyl administration (*P* > 0.05). No significant difference was observed regarding the HR and MAP among groups at each time point (Fig. 1).

Data of vital signs were collected at following time points: baseline (T0), after anesthesia induction (T1), at the beginning of the operation (T2), 10 min (T3) and 20 min (T4) after the surgery started, at the end of the surgery (T5), and after tracheal tube removal (T6).

**Fig. 2:**
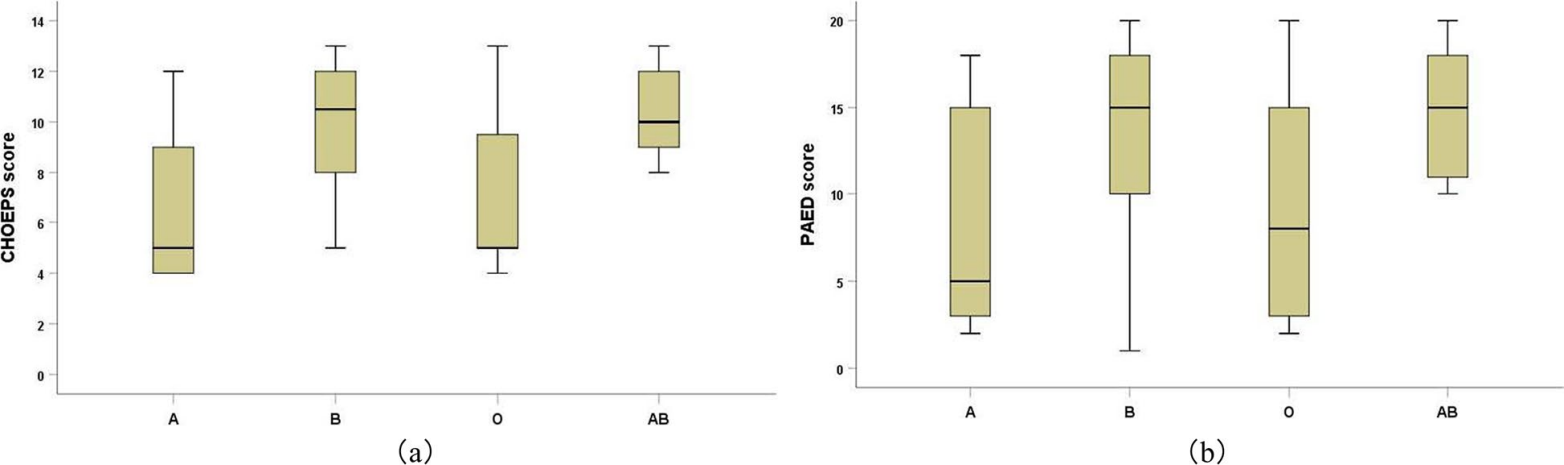
CHEOPS pain score (**a**) and PAED score (**b**) at the initial assessment in PACU (expressed as median and interquartile range). Both scores were significantly higher in Patients with AB and B blood type than those with A and O blood type (*P* < 0.001) CHEOPS, Children’s Hospital of Eastern Ontario Pain Scale. PAED, Pediatric Anesthesia Emergency Delirium

During initial pain assessment, patients in AB and B blood groups had significantly higher CHEOPS scores than those in A and O blood groups [10 (9–12) and 10.5 (8–12) vs. 5 (4–9.5) and 5 (5–10), *P*＜0.01](10 [9–12] and 10.5 [8–12] vs. 5 [4–9.5] and 5 [5–10], *P* < 0.01) (Fig. 2a). Overall, children in AB and B blood groups received significantly higher total doses of fentanyl over the PACU stay (*P* < 0.01), and a significantly higher percentage of patients in these two groups received fentanyl ≥ 1 μg/kg (*P* < 0.05) than that in the A and O blood groups (Table 2).

**Table 2:** Total PACU fentanyl requirement (average $\upmu$ g/person) and the number of patients who received fentanyl ≥ 1 μg/kg in the PACU among the groups

	A	B	O	AB	F	*P* value
Total PACU fentanyl(μg) Cases (%) ≥ 1 μg/kg	0.56 ± 0.62 11 (33.3)	1.07 ± 0.65 20 (66.7)	0.54 ± 0.55 11 (31.4)	1.12 ± 0.59 19 (73.1)	8.187 17.339	< 0.001 0.001

Patients in AB and B blood groups had significantly higher PAED scores than those in A and O blood groups [15 (10.75–18) and 15 (10–18) vs. 5 (3–15.5) and 8 (3–10), *P*＜0.01](15 [10.75–18] and 15 [10–18] vs. 5 [3–15.5] and 8 [3–10], *P* < 0.01) (Fig. 2b).

The patients in the AB and B blood groups had significantly longer PACU stay and a higher incidence of PONV within 24 h than those in other two blood groups (*P* < 0.001) (Table 3).

**Table 3:** The duration of PACU stay and the incidence of postoperative nausea and vomiting (PONV) over 24 h after surgery in the four groups

	A	B	O	AB	H-value	*P-value*
Duration of PACU(min)	20 (16–25)	30 (20–45)	20 (15–25)	30 (25–40)	30.392	< 0.001
PONV	1 (3.0)	14 (46.7)	2 (5.7)	13 (50.0)	32.278	< 0.001

*PACU*, post anaesthesia care unit, *PONV*, postoperative nausea and vomiting

## Discussion

This study was designed to investigate the association between ABO blood types and the postoperative pain. Our results showed that children in the AB and B blood groups had significantly higher CHEOPS pain scores in the PACU after T&A and subsequently required higher rescue fentanyl for pain management. Patients in AB and B blood types had significantly higher PAED scores than those in A and O blood types.

T&A is the first line of treatment for OSAS and is the most common procedure performed on children worldwide. Most children would develop moderate or severe post-T&A pain; however, the optimal and effective pain control approaches remain uncertain. Clinically, individual differences regarding pain thresholds and tolerance have been observed and many factors have been involved in the pain perception, including genetic traits [11]. Previous studies have investigated the relationship of pain with different genetic factors, such as sex and skin colour.

Our study results showed that paediatric patients in type AB and B blood groups had significantly higher CHEOPS pain scores after T&A and needed more rescue fentanyl for pain control in PACU than those in A and O blood groups. Amalie et al. [1] found that healthy volunteers with AB blood type showed increased sensitivity to mechanical pain stimulation of the craniofacial muscles and those with blood type B showed the lowest pain sensitivity (highest pain threshold). The partial discrepancy of results between Amalie’s and our study may be attributed to the differences in study design. Specifically, we observed the pharyngeal plexus, maxillary, and glossopharyngeal nerve with surgical stimulation in children, while Amalie et al.’s investigation observed the facial nerve with mechanical stimulation in adults. Lausten et al. [11] performed a retrospective investigation to examine the association between blood types and postoperative pain as indicated by the total consumption of postoperative analgesics, and they found no significant difference among ABO blood groups. We are unable to make further speculation about the differences of study conclusions since our study was prospective in children underwent T&A, vs theirs, retrospective in adults underwent knee surgery.

Effective pain control in children requires precise and valid pain assessment methods (12). The CHEOPS is a popular behavioral observational pain assessment scale developed by Mcgrath et al. in 1984 for measuring postoperative pain in children aged 1–7 years [13]. Multiple studies have selected a score of 6 on CHEOPS as the most appropriate indication for uncomfortable pain(14 -16), and our preliminary study also demonstrated that a score of 6 best presented the clinical situation, in which rescue fentanyl for pain should be initiated.

Many other factors could affect the individual’s pain perception, such as age, sex, weight or even surgical techniques. In study protocol, we kept a narrow age range and sex balance between groups, and excluded overweight and obese patients, and used a unique surgical technique to minimize the potential interference with the results.

Fentanyl has been a popular choice for its reliable sedative and analgesic effects during anaesthesia and surgery. Its onset of action is less than 60 s with a half-life of 90 min and duration of action of 30–60 min. Our results showed that the fentanyl administered during anaesthesia induction could effectively cover the entire surgical times (approximately 40 min) for all groups, and as fentanyl gradually worn off after patient arrived in PACU, patients in AB and B blood groups showed higher pain scores and required more rescue fentanyl afterwards.

Perioperative opioid use remains mainstream in pain management; however, their side effects have been a constant concern for patients and clinicians. In our study, children in AB and B blood groups showed higher CHEOPS pain scores and received more rescue fentanyl for pain control but PACU discharge had also been significantly delayed in those two groups as negative effect.

PONV, another most commonly undesirable side effect from anaesthesia, is involved in a variety of neurophysiological pathways, including central and peripheral receptor mechanisms (17) and the major risk factors are surgery, anaesthesia, as well as patient related [18]. One study showed that patients in the A blood group had higher susceptibility to nausea and vomiting during radiation therapy [19]. In our study, the AB and B blood groups had significantly higher incidence of PONV than the A and O blood groups did within 24 h post T&A. When all other clinical settings were similar among four ABO blood groups, we would consider the extra doses of rescue fentanyl as the culprit for the increased PONV in AB and B blood group.

Currently, there is no clear research evidence to explain what have exactly contributed to the higher postoperative pain score in AB and B blood group. It could be due to the difference in enzyme activity to affect the rate of opioid metabolism, or the difference in opioid receptor’s distribution and adaption in patients among ABO blood groups. Further studies are required to explore those hypothetical proposals and expected to shed light on this phenomenon of association between pain and ABO blood types.

There are limitations to our study that clinician should consider before any conclusions could be made. First, even though our patients had confirmed hypertrophic adenoids and tonsils presenting with airway obstructive symptoms, no sleep study (polysomnography) was conducted to grade the severity of OSAS prior to surgery. Reports have revealed that children with severe OSAS have significantly lower post-T&A opioid requirements than those with mild OSAS (20). However, performing a polysomnographic sleep study in every patient prior to T&A seems less practical in a busy SDS center by considering time-consuming and cost-ineffective matters. Secondly, four major ABO blood types are unequally distributed in the whole human population, which may skew the results to some degree. The distribution of AB, B, O, and A blood types, including Rh +/- is 5.5%, 16%, 45%, and 33.5%, worldwide respectively, and those numbers vary greatly among the countries. We tried to close the gaps of the total participants in each group, especially in AB blood type, to minimize potential statistical errors.

## Conclusion

Paediatric patients with AB and B blood types had higher postoperative CHEOPS pain scores and required significantly more rescue fentanyl to achieve comfortable pain control than those with A and O blood types after T&A. PAED scores in patients with AB and B blood types were also higher than those in patients with A and O blood type. Prolonged PACU stay and higher incidence of PONV ensued from more fentanyl consumption in AB and B group as the undesirable consequences of narcotics.

## Supplementary Information

**Additional file 1.**

**Additional file 2.**

## Data Availability

The datasets used and/or analyzed in the current study are attached in the Supplementary Materials section.

## Abbreviations

ASA: American Society of Anesthesiology
CHEOPS: Children’s Hospital of Eastern Ontario Pain Scale
PAED: Pediatric Anesthesia Emergency Delirium
PONV: Postoperative nausea and vomiting
T&A: Adenotonsillectomy
OSAS: Obstructive sleep apnoea syndrome
BIS: Bispectral index
