# Supplementary material for: Association between ABO blood groups and postoperative pain in children after adenotonsillectomy: a prospective cohort study
Source: BMC Anesthesiol. 2022 Dec 28;22:407. doi: 10.1186/s12871-022-01953-6 (PMC9795585; doi:10.1186/s12871-022-01953-6)
Supplement: Supplementary file 2 — Additional file 2. [file 12871_2022_1953_MOESM2_ESM.docx]

1.Groups 1, 2, 3 and 4 were A blood Group, B blood Group, O blood Group and AB blood Group respectively.

2.The M/F column is marked as 1 for male and 0 for female.

3.In PONV(postoperative nausea and vomiting) column, 1 is positive and 0 is negative. Interval time is the minimum time from induction fentanyl to the first rescue fentanyl.
